# Study on the Damage Regulation Mechanism of Low-Velocity Impact in CF/PA6 Laminates with Pre-Embedded Interlaminar Defect

**DOI:** 10.3390/polym18040436

**Published:** 2026-02-09

**Authors:** Fuwei Gu, Zhiyi Tian, Zhiyang Chen, Tianfeng Gi, Chengbo Ding

**Affiliations:** School of Mechanical Engineering, Jiangsu University of Technology, Changzhou 213001, China; 2024655117@smail.jsut.edu.cn (Z.T.); 2023655140@smail.jsut.edu.cn (Z.C.); 2025601054@smail.jsut.edu.cn (T.G.); 2025601032@smail.jsut.edu.cn (C.D.)

**Keywords:** low-velocity impact, pre-embedded defects, energy dissipation, damage regulation mechanism, melt-repair

## Abstract

Thermoplastic carbon fiber-reinforced polymer (CFRP) composites possess the intrinsic capability to heal delamination and matrix cracks via thermal re-melting. However, under impact loading, they are prone to severe fiber fracture, which significantly compromises their repairability. To address this, this study introduced polytetrafluoroethylene (PTFE) films as pre-set interlaminar defects within continuous carbon fiber-reinforced polyamide 6 (CF/PA6) thermoplastic cross-ply laminates. Low-velocity impact tests were conducted at varying energy levels to comparatively investigate the impact response and damage mechanisms of the CFRPs with and without embedded defects. Experimental results indicate that the embedded interlaminar defects triggered a transition in the failure mode of the CFRP from brittle fracture to progressive damage behavior. Compared to the baseline laminates, the specimens with embedded defects maintained higher flexural stiffness under low-energy impact. Furthermore, they effectively reduced the extent of fiber breakage by dissipating impact kinetic energy through extensive delamination, interlaminar frictional sliding, and plastic micro-deformation. These findings verify the feasibility of achieving macroscopic pseudo-ductility through interlaminar microstructural tailoring. This research provides an experimental basis and methodological support for the pseudo-ductile design of thermoplastic composites.

## 1. Introduction

Carbon fiber-reinforced polymer (CFRP) composites are indispensable in high-end equipment manufacturing due to their exceptional specific strength and stiffness [1,2]. In particular, thermoplastic CFRPs feature a remeltable matrix, endowing them with unique, inherent repairability. Realizing this potential relies on the active regulation of impact damage modes. Through interface design and structural optimization, impact energy can be strategically guided to dissipate via interlaminar delamination or matrix cracking. This mechanism mitigates irreversible fiber fracture while promoting repairable damage patterns. Consequently, investigating damage mode regulation to maximize repairability, alongside developing matching thermal repair technologies, constitutes a core strategy for enhancing the service safety and lifespan of thermoplastic CFRP components.

Conventional CFRP composites typically exhibit inherent linear elastic brittleness. Under low-velocity impact (LVI) loading, this brittleness results in matrix cracking, fiber fracture, and interlaminar delamination with negligible plastic deformation [3,4]. Such internal damage, often referred to as Barely Visible Impact Damage (BVID), significantly degrades the residual compressive strength of the structure and may even lead to catastrophic failure. However, by optimizing the fiber hybridization ratio, stacking sequence, and interface characteristics, the damage mode of composites can be regulated. This enables the laminates to undergo significant deformation and exhibit a gradual softening response without complete fracture, thereby maintaining load-bearing capacity. This phenomenon is termed pseudo-ductility [5,6]. The progressive failure characteristics of pseudo-ductile materials enhance structural reliability, potentially allowing for reduced design safety factors.

To endow brittle composites with such pseudo-ductile characteristics, multiphase hybridization has been proven to be the most direct and effective strategy. Researchers aim to create a yield plateau by introducing high-elongation components. For instance, Fang et al. [7] investigated the low-velocity impact (LVI) behavior of fiber-reinforced metal laminates. They confirmed that introducing a metal layer with excellent plastic deformation capacity significantly alters the failure mode, enabling the laminates to exhibit ductile characteristics similar to metals. Similarly, the evaluation of a unidirectional aramid/carbon fiber hybrid system by Liu et al. [8] demonstrated that the high toughness of aramid fibers can effectively inhibit the brittle fracture of carbon fibers, shifting the damage mode from localized penetration to large-area energy-absorbing deformation. Furthermore, Wang et al. [9] systematically compared carbon/glass fiber unidirectional and braided hybrid laminates to elucidate how the positive hybrid effect enhances impact resistance. Notably, they found that the braided structure limits the linear propagation of cracks via the mechanical interlocking of fiber bundles, thereby enhancing the impact toughness. To further transcend the limits of conventional materials, Wu et al. [10] recently designed a novel CFRP-rubber laminated structure. By utilizing the hyperelastic deformation capacity of the rubber layer as a stress buffer zone, they significantly reduced the peak impact force. This alternating design of rigid and compliant layers offers a new strategy for achieving pseudo-ductile energy absorption.

However, relying solely on the stacking of constituent materials is insufficient to achieve fully controllable pseudo-ductility; interlaminar properties and microstructure design are the governing factors determining the damage evolution path. Specifically, excessive interfacial bonding tends to cause brittle fiber fracture, whereas weak bonding results in premature delamination. Therefore, tailoring interlaminar behavior to achieve an optimal pseudo-ductile response is critical. The classical research by González et al. [11] indicated that the ply clustering effect directly alters the interlaminar shear stress distribution, thereby determining the initial threshold for delamination damage. Building on this, Zhang et al. [12] proposed an innovative layer thickness gradient design strategy, constructing a thick–thin–thick composite structure by symmetrically arranging ultra-thin and thick plies. This design successfully suppressed impact-induced delamination and induced a competitive mechanism between delamination and fiber fracture, thus significantly improving the impact resistance and residual compressive strength. Additionally, research by Zhang et al. [13] on interlaminar hybrid non-crimp fabrics (NCF) and the damage analysis of plain woven composites under multi-angle impact by Lv et al. [14] have confirmed that the interlaminar bridging effect can be enhanced by optimizing fabric structure and stitching technology, thereby demonstrating superior macroscopic damage tolerance.

It is worth noting that real-world impact scenarios are often more complex than standard laboratory tests. Consequently, the accumulation and interaction of damage impose higher demands on the pseudo-ductile impact toughness of materials. The core of pseudo-ductility lies in the capability to “work with damage.” Zhou et al. [15] investigated the positional effects of double impacts, revealing a critical issue: when impact points are spatially proximate, the stress fields in adjacent damaged areas overlap and couple, leading to a nonlinear accumulation of interlaminar damage. Under such complex loading conditions, damage propagation can easily exceed the toughness limit of a single material, resulting in the failure of the pseudo-ductile mechanism. Furthermore, Ding et al. [16] examined the effects of impactor geometries, while Chen et al. [17] analyzed the damage evolution mechanism in specimens with prefabricated defects. Their findings clarify at the microscopic level that without accurate control over the evolution of initial interlaminar defects under complex stress states, the global ductile response of the structure cannot be guaranteed. Huang et al. [18] revealed that the pseudo-ductile behavior of carbon/glass fiber hybrid composites exhibits a significant size effect. Increasing the width leads to premature delamination due to the expanded interfacial area, thereby degrading the quality of the pseudo-ductile yield plateau. Conversely, increasing the thickness significantly weakens the ultimate load-bearing capacity due to the intensified complexity of internal stress gradients.

In summary, although existing literature has achieved significant results in hybrid modification and macroscopic energy absorption evaluation, critical limitations remain. Most current designs primarily focus on enhancing the peak load or total energy absorption of materials. However, a key unresolved problem in this field is how to introduce a mechanism at the interlaminar interface level to achieve a gradual damage evolution. By facilitating regulated interlaminar slip or delamination under impact, such a mechanism aims to minimize fiber fracture while simultaneously balancing impact pseudo-ductility and structural integrity.

Continuous carbon fiber and thermoplastic matrix combinations have evolved into various processing variants to overcome the impregnation barriers caused by high melt viscosity, including melt-calendared unidirectional (UD) tapes, comingled yarns using fiber hybridization, film stacking for lamination, and powder-impregnated tows [19]. Among these matrix systems, Polyamide 6 (PA6) stands out due to its superior balanced performance. As a typical semi-crystalline polymer, PA6 not only demonstrates excellent flowability and processability but also exhibits fracture toughness and impact resistance far superior to traditional thermosetting resins [20]. Its high molecular chain ductility enables the matrix to dissipate energy through extensive plastic yielding under impact loads. Furthermore, the abundant polar amide groups in the PA6 chain facilitate good interfacial bonding with the carbon fiber surface, while its inherent thermoplasticity grants the material the ability to re-melt upon heating, providing a solid physical basis for secondary forming, post-impact thermal repair, and recycling [21].

This study aims to enhance the impact resistance of composite laminates by proposing a thermoplastic carbon fiber composite laminate with interlaminar defects, with a focus on the contribution mechanism of interlaminar defects to impact pseudo-ductility. Given that the thermoplastic matrix can be remolded upon heating, the larger interlaminar damage interface induced by pseudo-ductility, which preserves fiber continuity, may offer potential for subsequent thermal repair of the material. However, the specific performance after repair requires systematic investigation. Therefore, reparability is identified as a clear direction for future research. Low-velocity impact tests were conducted to compare the impact resistance of carbon fiber thermoplastic composite laminates without delamination defects and those with such defects. Using defect-free CFRP laminate specimens as a reference, low-velocity impact tests were performed at three energy levels—10 J, 20 J, and 30 J. The impact response behavior of the laminates was thoroughly analyzed, and ultrasonic C-scanning was employed to investigate the impact resistance and damage modes of CFRP laminates with delamination defects. This study provides experimental evidence and methodological support for the design of pseudo-ductility in thermoplastic composites.

## 2. Materials and Methods

### 2.1. Preparation of Composite Laminates with and Without Pre-Embedded Defects

The materials used in this experiment were unidirectional carbon fiber/PA6 prepregs, with a resin content of approximately 35% and a nominal thickness of 0.16 mm. In accordance with the ASTM D7136/D7136M standard, the dimensions of both laminates were 150 mm × 100 mm, with a stacking sequence of [(0°/90°)_9_]. Hereinafter, C0 denotes the laminate without pre-embedded defects, and C1 denotes the laminate with pre-embedded defects. The specific geometric parameters are shown in Table 1.

**Figure 1:**
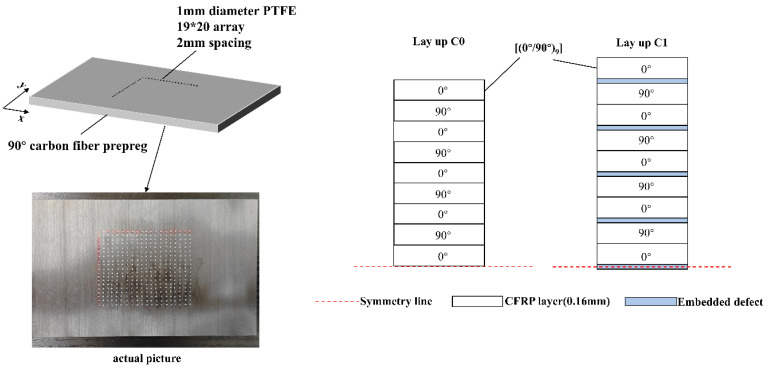
Placement configuration of pre-embedded defects and laminate stacking sequence.

Given the brief contact duration of low-velocity impacts, the spacing between defects was designed to minimize the distance for stress wave propagation. Excessive spacing would result in a sparse defect distribution, failing to effectively guide the compressive stress waves between layers. Therefore, a specific defect interval was selected to facilitate rapid crack extension and growth within the short impact contact time. When the laminate is subjected to an external impact load, these cracks propagate continuously within the interlayers, thereby absorbing impact energy and increasing the delamination area. Defects were obtained by punching Polytetrafluoroethylene (PTFE) film with a 1 mm diameter puncher. The defects were arranged in a lattice pattern and placed manually on each 90° unidirectional carbon fiber prepreg layer. PTFE film with a thickness of 0.08 mm was selected as the defect material. The film exhibits a temperature resistance range of −196 °C to 260 °C, which is sufficient to withstand the molding temperature without decomposition. The defects had a diameter of 1 mm and were spaced at intervals of 2 mm. There were 19 defects arranged along the fiber direction and 20 perpendicular to the fiber direction, resulting in a total of 380 defects. The total defect area was 298.45 mm^2^, corresponding to an interlayer defect area fraction of 1.04% in the composite laminates with pre-embedded defects. The process of placing interlayer defects in the composite laminates is illustrated in Figure 1.

**Figure 2:**
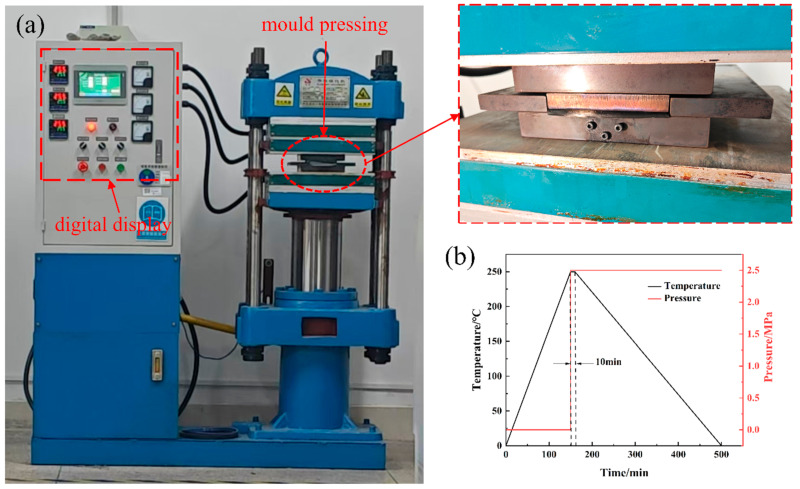
(**a**) Sample preparation using a flat vulcanizer and (**b**) molding temperature and pressure curves.

The laminates were fabricated using an XLB-D400 × 400 × 2/0.50 MN column-plate vulcanizing press (Shanghai, China). Initially, the temperature was raised to the molding temperature of 250 °C. The mold was then closed and pressurized to 2.5 MPa, and this condition was maintained for 10 min. Subsequently, the heating system was deactivated, allowing the laminate to cool slowly to room temperature. The detailed preparation process is illustrated in Figure 2.

### 2.2. Low-Velocity Impact Test

**Figure 3:**
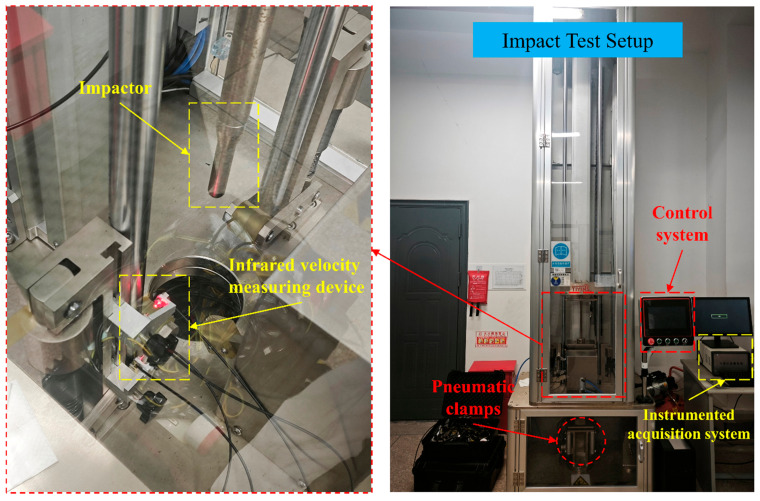
Drop weight impact testing machine.

Low-velocity impact tests were conducted on the carbon fiber composite laminates using a FIT7522–1 instrumented drop-weight system (Shanghai ailadi industry technology Co., Ltd. Shanghai, China), in accordance with the ASTM D7136 standard. As shown in Figure 3, the experimental setup consisted of a pneumatic clamping module, guide columns, a hemispherical impactor with a diameter of 16 mm and a weight of 14.853 kg and a dynamic signal acquisition system with a maximum sampling frequency of 2 MHz. The clamping fixture was composed of upper and lower rectangular steel plates with central cutouts. During the test, the specimen was secured between the rectangular supports using the pneumatic fixture. Impact energies of 10 J, 20 J, and 30 J were achieved by adjusting the drop height. Table 2 shows the impact parameters of the test. Prior to testing, the specimen position was calibrated to ensure alignment between the center of the impactor and the center of the specimen. A piezoelectric force sensor and an infrared velocimeter were used to synchronously acquire the load-time history and impact velocity data. Finally, a pneumatic lifting mechanism was employed to retract the impactor immediately after the initial impact to prevent secondary impacts. The absorbed energy ($E_{ab}$) in this study was obtained by applying trapezoidal numerical integration (Δd = 0.001 mm) to the load–displacement curve.

(1) $$E_{ab}=\int_{0}^{d_{\max}}F(d)dd$$

where: F(d) represents the contact force; $d_{\max}$ represents the maximum displacement of the impactor.

## 3. Results and Discussion

### 3.1. Force–Time Response

**Figure 4:**
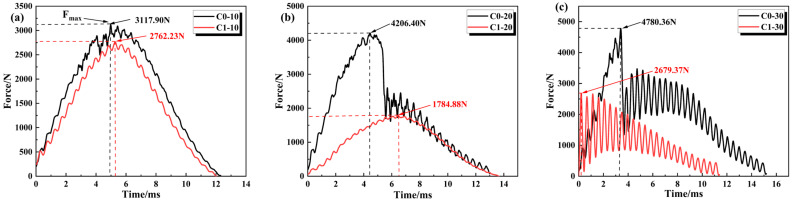
Force–time curves of CFRP laminates with and without pre-embedded defects under impact energies of (**a**) 10 J, (**b**) 20 J, and (**c**) 30 J.

Figure 4a–c illustrates the force–time response curves of CFRP samples with and without defects under different impact energies. In the initial loading stage, the impact force increases almost linearly, with the impact energy primarily absorbed by the elastic compression of the topmost CFRP plies. As the impact time increases, the curve begins to fluctuate after reaching the peak force, indicating the initiation of accumulated damage within the sample. In the unloading stage, the impactor rebounds, and the impact force gradually decreases to zero, during which the sample dissipates energy through progressive damage propagation. Specifically, under the low-energy impact of 10 J, both materials exhibit typical semi-sinusoidal pulse characteristics with an effective contact duration of approximately 12.5 ms. However, the C1 sample displays a significant peak truncation effect; its peak load decreases to 3150 N (a reduction of approximately 12.7% compared to C0). This indicates that the presence of defects alters the energy dissipation pathway, compelling the material to dissipate more energy through internal deformation or enhanced damping mechanisms.

With the impact energy increasing to 20 J, the failure modes of the two materials diverged significantly. The C0 sample exhibited an initial peak load as high as 4150 N; however, at 5.5 ms, a sudden load drop with an amplitude exceeding 40% occurred, indicating the onset of macroscopic brittle failure such as matrix cracking or fiber fracture. In contrast, although the peak force of the C1 sample decreased to 1750 N, it displayed a smooth and continuous response curve without any catastrophic sudden drop. This suggests that C1 maintained good structural integrity during impact, relying primarily on elastic-plastic deformation rather than brittle fracture to absorb energy. It is worth noting that the above phenomenon is based on the representative response curve, and its stable rebound behavior and related trends still need to be confirmed by further statistical verification.

However, under the condition of 30 J high–energy impact, the limitations of current defect configuration become obvious. When the C0 sample rose linearly to 4800 N, and then catastrophic macroscopic fracture occurred, the C1 sample showed violent oscillation from the beginning, because the excessive impact energy brought large-area delamination damage and partial fiber fracture, which led to the stiffness of the laminated plate being unbalanced from the beginning, the peak load was significantly attenuated to 2700 N, and the curve showed zigzag nonlinear oscillation. These fluctuations are caused by excessive stress concentration at defects, which shows that although the design improves the damage tolerance at low energy, the current defect rate and spacing need to be further optimized to withstand such high-energy impact as 30 J without damaging the structural integrity.

In the force–time curve, the first peak corresponds to the elastic response and initial damage of the topmost CFRP plies. At this stage, the load-bearing capacity of the material interface and matrix is temporarily compromised due to cracking. In the C1 sample, the presence of interlaminar defects facilitates rapid crack propagation along the interlaminar interfaces of the laminates, which consequently reduces the overall load-bearing capacity of the specimen.

### 3.2. Force–Displacement Response

**Figure 5:**
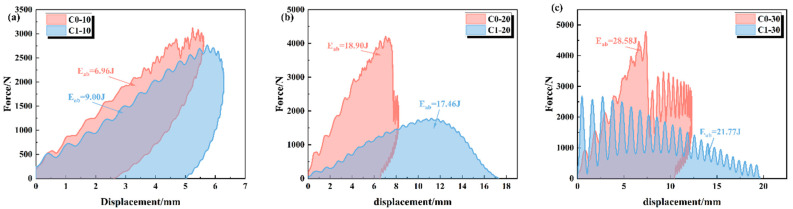
Force–displacement curves of CFRP laminates with and without pre-embedded defects under impact energies of (**a**) 10 J, (**b**) 20 J, and (**c**) 30 J.

Figure 5 illustrates the force–displacement response curves of CFRP specimens without and with embedded defects under different impact energies. In general, both curves exhibit obvious serrated characteristics during the loading phase, which corresponds to the progressive accumulation of local damage, such as matrix cracking or local crushing within the structure.

Figure 5a clearly demonstrates that the presence of internal defects shifts the failure mode of CFRP laminates from the brittle–elastic–dominated mode of C0 to the pseudo-ductile–dominated mode of C1. The comparison reveals that the peak load of the C1 sample is maintained at a high level under 10 J impact; it is only approximately 5% lower than that of C0 (which is about 3100 N), indicating that the defects did not lead to a catastrophic loss of load-bearing capacity. More importantly, there are essential differences in the unloading stage: C0 exhibits a typical elastic rebound with a residual displacement of only 2.6 mm, whereas C1 displays a nearly vertical unloading path. This remarkable hysteretic behavior and large residual deformation confirm that the C1 sample possesses mechanical characteristics similar to ductile materials; that is, it absorbs impact energy by utilizing nonlinear strain induced by internal defects. This pseudo-ductility mechanism enables the structure to avoid brittle fracture by allowing a certain degree of deformation under non-catastrophic impact conditions.

With the impact energy increasing to 20 J and 30 J (Figure 5b,c), the mechanical response mechanisms of the two materials diverged fundamentally. The C0 sample exhibits typical high-strength and brittle characteristics: its peak load is extremely high, but upon reaching a critical displacement, the load drops vertically. This indicates catastrophic failure, such as fiber fracture or penetration, leaving the sample with almost no subsequent load-bearing capacity. In contrast, the C1 sample presents a unique mechanism of low-strength progressive damage. Although its peak load is significantly lower than that of C0, it demonstrates excellent ductility and damage tolerance. At 20 J, the curve shows a broad arch feature, with the effective compression displacement extending to 17.5 mm. At 30 J, the curve further evolves into violent periodic oscillations, accompanied by extensive displacement collapse and no obvious rebound. The remarkable characteristics of load oscillation and large displacement demonstrate that the defect-induced material behavior transforms from instantaneous brittle fracture to gradual collapse, realizing energy dissipation through continuous local microstructural failure.

### 3.3. Energy–Time Response

**Figure 6:**
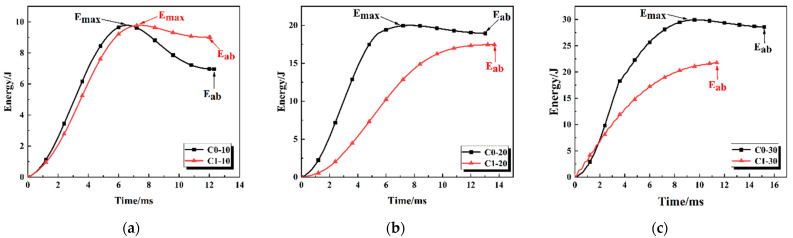
Energy–time curves of CFRP laminates with and without pre-embedded defects under impact energies of (**a**) 10 J, (**b**) 20 J, and (**c**) 30 J.

Figure 6 illustrates the energy–time response curves of CFRP samples without (C0) and with (C1) embedded defects under different impact energies. As observed in Figure 6a, under an impact energy of 10 J, both samples reach a similar peak energy of approximately 9.8 J, which corresponds to the moment of maximum deformation during the impact process. However, significant differences exist in the post-impact rebound stage. After C0 reaches its peak, the energy curve exhibits a distinct drop, finally stabilizing at about 7.0 J. In contrast, the energy curve of C1 shows minimal decrease after the peak, with the final energy absorption value stabilizing at approximately 9.0 J. The negligible rebound energy of C1 indicates that most of the impact kinetic energy is effectively dissipated through plastic deformation or internal damage. Conversely, the C0 specimen demonstrates a typical elastic-dominated response mechanism, where most of the input kinetic energy is converted into reversible elastic potential energy and stored in the laminate, corresponding to the minimal residual deformation observed in its force–displacement curve. As shown in Figure 6b, C0 exhibits a rapid energy absorption characteristic; its energy curve rises sharply in the initial stage, reaching a peak of about 20 J at approximately 7 ms. This suggests that the structure possesses high contact stiffness and can rapidly decelerate the impactor. On the contrary, the energy absorption process of C1 is more gradual and delayed. The energy curve rises with a lower slope, reaching its maximum value after about 13 ms, and the final absorbed energy is slightly lower than that of the C0 sample. This extended response time demonstrates that the C1 sample provides a superior buffering effect, dissipating energy gradually by prolonging the impact duration. Regarding the 30 J high-energy impact in Figure 6c, the energy curve of C0 rises rapidly, reaching a peak of about 30 J at approximately 9.5 ms. Subsequently, the curve drops slightly, indicating that most of the energy was permanently absorbed by the structure (due to catastrophic failure). Although the energy absorption rate of C1 is similar to C0 in the initial 0–2 ms stage, the energy growth decelerates significantly during the subsequent main deformation stage, finally reaching a maximum energy absorption of only about 22 J.

**Figure 7:**
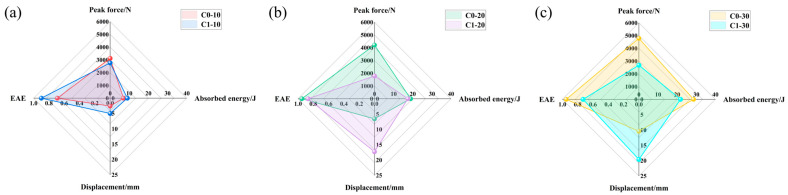
Performance comparison radar charts of CFRP laminates with and without pre-embedded defects under impact energies of (**a**) 10 J, (**b**) 20 J, and (**c**) 30 J, showing peak force, absorbed energy, impact displacement, and Energy Absorption Efficiency (EAE).

Figure 7 presents radar charts illustrating the peak force, displacement, and energy absorption efficiency (EAE) of the specimens under various impact energies. The EAE can be calculated by Formula (2). As the impact energy increased, the peak force of the C0 specimens exhibited a continuous upward trend. In contrast, the C1 specimens experienced a load drop at 20 J and 30 J due to severe internal fiber breakage, indicating that they had reached their load-bearing limit. Specifically, under 10 J impact, the pre-embedded delamination defects induced rapid crack propagation and reduced structural stiffness, resulting in an 11.4% decrease in the peak force of C1 compared to C0. It is worth noting that this stiffness degradation endowed the C1 specimens with significant structural “pseudo-ductility.” The defects disrupted the interlayer continuity, leading to severe non-linear large deformation under impact loads. The data indicates that at impact energies of 10 J, 20 J, and 30 J, the maximum displacement of C1 surged by 93.1%, 163.3%, and 88.1%, respectively, compared to C0. This reflects that when the external force exceeded the elastic limit, the interlayer sliding induced by the defects accelerated the accumulation of irreversible deformation. This unique failure mode had a dual effect on the energy absorption characteristics. Under the 10 J low-energy impact, the C1 specimens benefited from the enhanced energy dissipation caused by crack propagation, resulting in a 29% increase in energy absorption compared to C0. However, under the 20 J and 30 J high-energy impacts, the excessive softening associated with the pseudo-ductility led to premature penetration failure. This conversely restricted their ultimate energy absorption capacity, resulting in reductions of 8% and 24% compared to C0, respectively.

(2) $$EAE=\frac{E_{A}}{E_{I}}$$

where: $E_{A}$ represents the absorbed energy, $E_{I}$ represents the impact energy.

### 3.4. Damage Morphology Analysis

**Figure 8:**
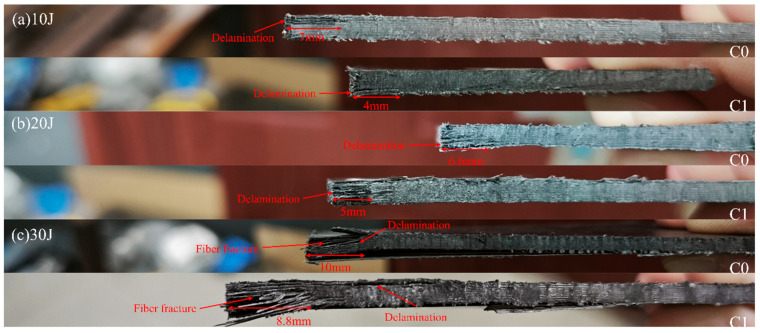
Side-view damage morphology of CFRP specimens with and without pre-embedded defects under different impact energies.

**Figure 9:**
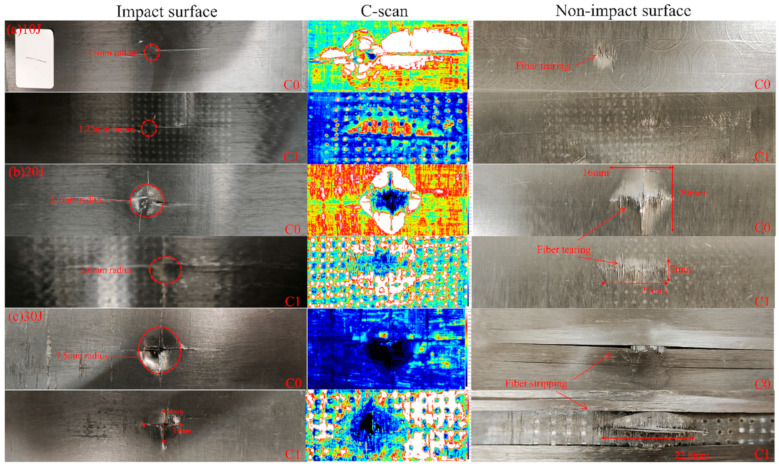
Damage morphology of the impact and non-impact sides, and C-scan images of the central impact region of CFRP specimens with and without pre-embedded defects under different impact energies. (Both white and red parts represent delamination defects, but the delamination damage degree of the two parts is different, and the delamination damage degree of the white part is greater).

Figure 8 displays the side-view damage morphology of CFRP laminates without (C0) and with (C1) interlaminar defects after sectioning under different impact energies. As observed in Figure 8, due to its high stiffness, the defect-free C0 sample releases stress primarily through extensive interlaminar crack propagation (reaching 7 mm and 6.6 mm at 10 J and 20 J, respectively) and severe fiber fracture. In contrast, the interlaminar defects in the C1 sample provide a pathway for energy release. Upon impact, the majority of the impact energy is preferentially dissipated by driving crack propagation within the defect regions; this process consumes energy, thereby mitigating excessive stress concentration on the fibers. This mechanism—prioritizing matrix damage for energy dissipation—successfully alleviates impact damage to the fiber layers. Consequently, C1 dissipates energy through defect expansion, which not only confines damage to a more localized region (crack lengths of only 4–5 mm) but also significantly reduces the severity of fiber fracture (as corroborated by Figure 9), thus avoiding the catastrophic brittle failure observed in C0.

Figure 9 illustrates the comparison of damage evolution between C0 and C1 under different impact energies. Experimental results demonstrate that the embedded interlaminar defects fundamentally alter the damage propagation path and energy dissipation mechanism. Quantitative analysis was performed using Fiji software (Image J 1.54p) to measure the projected delamination area (based on a defined signal threshold) and using vernier caliper to measure the surface indentation dimensions.

C-scan results reveal a distinct trade-off mechanism. At 20 J and 30 J, the C1 samples exhibited significantly larger delamination areas (347.30 mm^2^ and 451.54 mm^2^, respectively) compared to C0 (295.81 mm^2^ and 262.35 mm^2^). This grid-like expansion in C1 confirms that the defects successfully acted as stress concentration initiators, guiding cracks laterally to mobilize a larger material volume for energy dissipation.

This lateral dispersion of energy significantly protected the structural integrity of the impact zone. Measurements of the impact pits reveal that C1 consistently maintained smaller indentation radii than C0. At 10 J and 20 J, the indentation radii for C0 were 1.5 mm and 5.1 mm, whereas C1 showed reduced values of 1.25 mm and 3.6 mm, respectively. This indicates that by promoting internal delamination, the C1 design alleviates local contact stresses.

The contrast is most critical under the 30 J high-energy impact. The C0 sample suffered a deep, circular indentation with a radius of 7.5 mm, accompanied by a counterintuitive decrease in delamination area (262.35 mm^2^). This decrease signals a transition to catastrophic failure: instead of delaminating, the material was perforated. In stark contrast, the C1 sample developed a shallower, elliptical indentation (14 mm × 11 mm, with the major axis perpendicular to the fiber direction), indicating that the defects successfully diverted the damage path away from the principal fiber orientation.

Visual inspection of the non-impact side (back face) further corroborates the protective role of the defects. Across all energy levels, the back-face damage of the C0 samples was visibly more severe than that of C1. While C0 exhibited extensive fiber peeling, breakage, and localized perforation (especially at 30 J), the C1 samples maintained better fiber continuity with damage primarily restricted to the interlaminar interfaces. This conclusively proves that the ‘multi-point’ blooming mode of C1 effectively sacrifices internal interface bonding to prevent catastrophic fiber fracture and through-thickness penetration, thus protecting the fiber and maintaining structural integrity.

## 4. Conclusions

Addressing the problem of brittle fracture in conventional carbon fiber-reinforced polymer (CFRP) laminates under low-velocity impact (LVI), this paper proposes a composite laminate with embedded interlaminar defects and investigates its contribution mechanism to pseudo-ductility via LVI tests and post-impact ultrasonic C-scanning. The experimental results show that the design successfully realizes the transition from brittle failure to progressive failure mode within the effective impact energy range (10–20 J). Compared with the benchmark laminates, the laminates with embedded interlaminar defects maintain high flexural stiffness while significantly improving damage tolerance, validating the feasibility of achieving macroscopic pseudo-ductility by regulating the interlaminar microstructure.

1. Improvement of impact resistance: The results of 10 J impact tests indicate that the energy absorption efficiency (EAE) of the C1 laminate increased by 29%, while the peak impact force decreased by 12.7%, and the maximum displacement increased by 93.1%. This demonstrates that the embedded defects effectively act as stress buffers and energy dissipation mechanisms, thereby enhancing the impact resistance of the CFRP laminates.
2. Pseudo-ductile failure mechanism: The energy absorption mechanism of laminates with embedded interlaminar defects differs distinctively from that of reference laminates (which rely primarily on matrix cracking and fiber fracture). C1 dissipates a substantial amount of impact kinetic energy through extensive interlaminar frictional slip and plastic micro-deformation. Analysis of the impact curves reveals that the interlaminar interface of C1 successfully transforms instantaneous brittle fracture into a multi-stage progressive failure. The impact load first triggers controlled interlaminar delamination, subsequently inducing fiber bridging and pull-out. This delamination–bridging synergy effectively delays crack propagation into deeper layers, manifesting as a smooth unloading phase rather than a sudden load drop.
3. While the proposed design demonstrates significant improvements in damage tolerance and energy dissipation under low-energy impact (10–20 J), it is essential to address the structural and manufacturing trade-offs associated with the insertion of PTFE layers. Firstly, regarding manufacturing and geometry, the insertion of 0.08 mm PTFE films introduces a discrete increase in laminate thickness. In practical applications, this parasitic weight and volume must be weighed against the benefits of impact protection. However, this issue can be mitigated in future iterations by employing thinner interleaving materials (e.g., nanofiber veils or sprayed coatings) rather than commercial PTFE films, which would minimize the thickness penalty while maintaining the de-bonding mechanism. Secondly, regarding mechanical performance, the weak bonding interface provided by PTFE, which is the key to the crack-arresting mechanism during high-speed impact, inevitably compromises the interlaminar shear strength (ILSS) and static in-plane properties. We acknowledge that this design creates a trade-off: ensuring structural continuity under static loads versus enabling sacrificial delamination under impact. Therefore, this defect-engineered configuration is not intended as a universal replacement for standard laminates but rather as a specialized solution for components specifically exposed to high threat of impact, such as ballistic armor or crash-absorbing automotive structures.
4. Limitations and Future Work: In this paper, the influence of embedded defects on the overall stiffness and static strength of laminated plates has not been evaluated. Moreover, due to the repeatability caused by artificial laying defects and the limitation of statistical evaluation, systematic statistical verification will be carried out in future research. In addition, thermoplastic-based materials offer potential possibilities. The future work will be devoted to exploring a balanced design method by optimizing the defect distribution, aiming at maximizing the damage tolerance without significantly sacrificing the structural stiffness and strength, and evaluating the residual strength of the laminated plate after thermal repair, so as to fully tap the versatility of this design.

## Figures and Tables

**Table 1:** Geometric parameters of sample.

Specimen	Thickness/mm
C0	2.46
C1	2.62

**Table 2:** Impact test parameters.

Energy/J	Velocity/(mm·s^−1^)	Height/mm
10	1160	68.7
20	1641	137.4
30	2010	206.1

## Data Availability

The data presented in this study are openly available at https://pan.baidu.com/s/1YncBoyJmjnXyfhwQEhvgMw?pwd=7czv (accessed on 27 January 2026).
